# Targeted inhibition of metastatic melanoma through interference with Pin1-FOXM1 signaling

**DOI:** 10.1038/onc.2015.282

**Published:** 2015-08-17

**Authors:** F Kruiswijk, S C Hasenfuss, R Sivapatham, M P Baar, D Putavet, K A T Naipal, N J F van den Broek, W Kruit, P J van der Spek, D C van Gent, A B Brenkman, J Campisi, B M T Burgering, J H J Hoeijmakers, P L J de Keizer

**Affiliations:** 1 Center of Molecular Medicine, Molecular Cancer Research, University Medical Center, Utrecht, The Netherlands; 2 The Buck Institute for Research on Aging, Novato, CA, USA; 3 Department of Genetics, Erasmus Medical Center Rotterdam, Rotterdam, The Netherlands; 4 Department of Medical Oncology, Erasmus Medical Center Rotterdam, Rotterdam, The Netherlands; 5 Department of Bioinformatics, Erasmus Medical Center Rotterdam, Rotterdam, The Netherlands; 6 Department of Metabolic and Endocrine Diseases, University Medical Center, Utrecht, The Netherlands; 7 Life Sciences Division, Lawrence Berkeley National Laboratory, Berkeley, CA, USA

## Abstract

Melanoma is the most lethal form of skin cancer and successful treatment of metastatic melanoma remains challenging. BRAF/MEK inhibitors only show a temporary benefit due to rapid occurrence of resistance, whereas immunotherapy is mainly effective in selected subsets of patients. Thus, there is a need to identify new targets to improve treatment of metastatic melanoma. To this extent, we searched for markers that are elevated in melanoma and are under regulation of potentially druggable enzymes. Here, we show that the pro-proliferative transcription factor FOXM1 is elevated and activated in malignant melanoma. FOXM1 activity correlated with expression of the enzyme Pin1, which we found to be indicative of a poor prognosis. In functional experiments, Pin1 proved to be a main regulator of FOXM1 activity through MEK-dependent physical regulation during the cell cycle. The Pin1-FOXM1 interaction was enhanced by BRAF^V600E^, the driver oncogene in the majority of melanomas, and in extrapolation of the correlation data, interference with\ Pin1 in BRAF^V600E^-driven metastatic melanoma cells impaired both FOXM1 activity and cell survival. Importantly, cell-permeable Pin1-FOXM1-blocking peptides repressed the proliferation of melanoma cells in freshly isolated human metastatic melanoma *ex vivo* and in three-dimensional-cultured patient-derived melanoids. When combined with the BRAF^V600E^-inhibitor PLX4032 a robust repression in melanoid viability was obtained, establishing preclinical value of patient-derived melanoids for prognostic use of drug sensitivity and further underscoring the beneficial effect of Pin1-FOXM1 inhibitory peptides as anti-melanoma drugs. These proof-of-concept results provide a starting point for development of therapeutic Pin1-FOXM1 inhibitors to target metastatic melanoma.

## Introduction

Metastatic melanoma is the most lethal type of skin cancer with an average survival rate of 8–18 months when untreated.^1, 2^ Treatment options mainly consist of immunotherapy, or targeted therapies against activated oncogenic pathways, both of which have limitations. Immunotherapy does provide a prolonged clinical response, but is mainly effective in a subset of patients.^3^ Targeted therapies are generally designed around inhibition of the pro-proliferative kinase MEK. MEK is constitutively activated in the vast majority of all melanomas due to activating mutations in the upstream kinases BRAF or NRAS, with V600E-mutated BRAF being the oncogenic driver of ~50% of all melanomas.^4^ Repression of mutated BRAF or MEK proved to strongly reduce the growth of several melanomas.^5, 6^ Indeed, small molecule inhibitors against mutant BRAF such as Vemurafenib and Dabrafenib provide a potent initial clinical benefit and delay, but not prevent, patient mortality.^7, 8^ Unfortunately, additional mutations in the same or parallel pathways occur rapidly, keeping MEK activity high and the overall survival rate low.^9^

To improve patient survival, new therapies would either have to enhance initial drug efficacy, repress acquired drug resistance or inhibit downstream targets of MEK in an alternative manner. We focused on the latter approach by searching for new druggable weak spots in malignant melanoma.

## Results

### FOXM1 is elevated and active in melanomas

**Figure 1:**
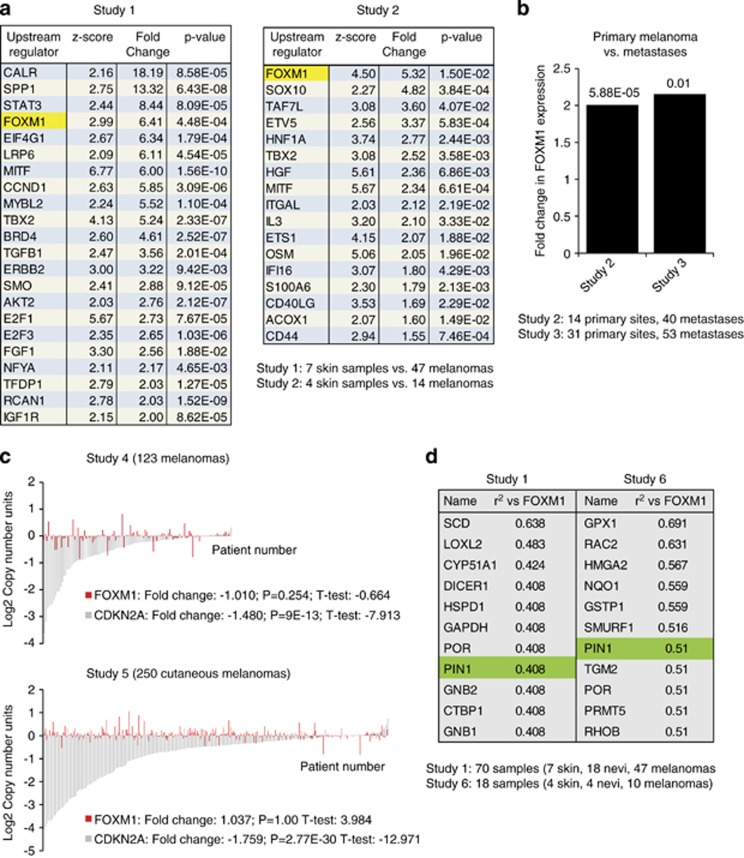
FOXM1 is elevated and activated in malignant melanoma. (**a**) Ingenuity Pathway Analysis (IPA; Version build 242990) for upstream regulators in data sets obtained from Oncomine.^43, 44^ Shown are those genes predicted to be activated based on upstream regulator analysis (*z*-score) and that are themselves upregulated at least 1.5-fold with a *P-*value <0.05. (**b**) Expression of FOXM1 mRNA in two independent Oncomine data sets,^44, 45^ comparing primary melanoma vs metastases. *P-*values are indicated. (**c**) Analysis of copy number variation of FOXM1 in two independent Oncomine data sets^46^ (and The Cancer Genome Atlas melanoma). CDKN2A is shown as a control and is known to show a reduction in copy number in many melanomas.^15^ (**d**) Co-expression analysis for genes correlating with FOXM1 in two independent Oncomine data sets,^43, 47^ showing the top candidates to co-express with FOXM1 in melanoma.

We initiated this study by performing a database analysis to identify pro-proliferative and pro-survival factors that are elevated in melanoma. MEK is chronically activated in the majority of melanomas, and MEK activation is a prime cause of resistance to BRAF inhibitors.^10^ Therefore, we focused on factors that are under potential regulation of MEK signaling as we reasoned these could be potential candidates for therapeutic intervention of melanomas resistant to BRAF/MEK inhibitors. We used Ingenuity Pathway Analysis on gene expression profiles from independent data sets to identify molecular pathways that are activated in melanoma compared with normal skin. One hit that was both projected to be active by Ingenuity Pathway Analysis and was also elevated in melanoma was FOXM1 (Figure 1a), a MEK target.^11^ We found FOXM1 to correlate with progressive disease status (Figure 1b), suggesting FOXM1 may be relevant to melanoma development. FOXM1 is a transcription factor that is expressed and activated during active cell cycle progression,^12^ further underscoring a potential role in tumor progression, and FOXM1 has been implicated in the chemoresistance of other types of cancer.^13, 14^ We thus set out to study whether FOXM1 could be a suitable target of intervention against melanoma.

First, we analyzed whether the increase in FOXM1 expression in melanomas could be due to gene amplification. In contrast to CDKN2A, which is known to be lost in many melanomas,^15^ we did not observe significant changes in FOXM1 gene copy number (Figure 1c), suggesting the FOXM1 increase may be due to elevated transcription.

FOXM1 is known to regulate its own transcription in a feed-forward fashion.^16, 17^ As FOXM1 activation could therefore be responsible for elevations in its messenger RNA (mRNA) expression, we next searched for genes correlating with FOXM1 expression. We specifically focused on enzymes, reasoning that they might be upstream regulators of FOXM1 activity and hence candidates for therapeutic intervention. Co-expression analysis of two independent melanoma data sets revealed several potential enzymatic regulators of FOXM1. One enzyme, which convincingly appeared in both data sets, was Pin1 (Figure 1d).

### Pin1-FOXM1 signaling is elevated in metastatic melanoma and indicative of poor disease outcome

**Figure 2:**
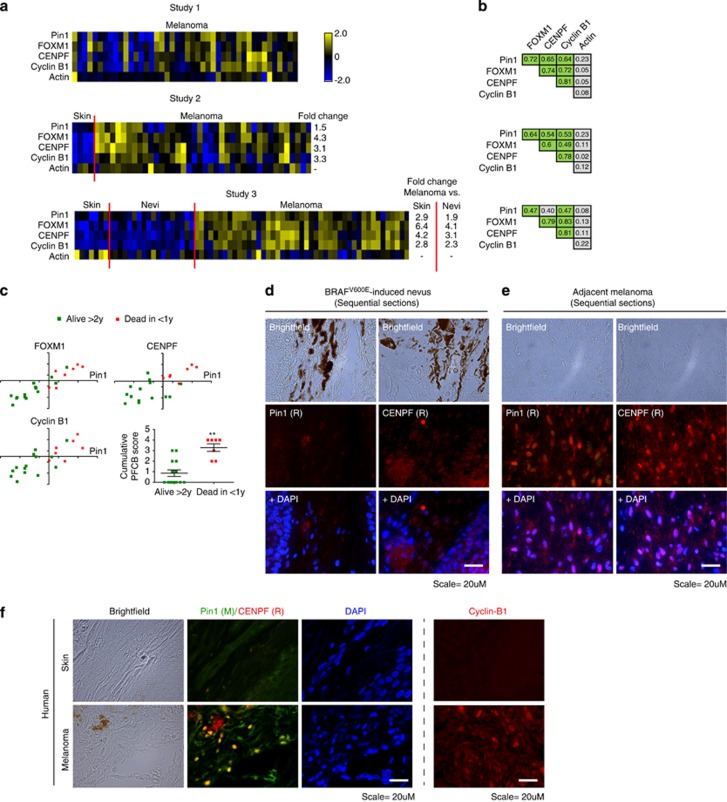
FOXM1, CENPF, Cyclin B1 and Pin1 mRNA levels correlate in malignant melanoma and associate with poor disease outcome. (**a**) Heatmaps showing relative *z*-score corrected mRNA expression profiles of Pin1, FOXM1, CENPF, Cyclin B1 and actin in three independent Oncomine data sets.^43, 44, 48^ Where applicable, the fold difference between normal skin, benign nevi and melanoma are indicated, all of which are significant (*P*<0.01). (**b**) Overview of correlation coefficients for the indicated gene expression changes from the data sets in A. Green indicates a correlation of *R*>0.45; *P*<0.01. (**c**) Scatter plots of a selection of the Bogunovic dataset^48^ from Oncomine showing correlation between Pin1 and FOXM1, CENPF and Cyclin B1 subdivided into patient groups that lived >2 years or <1 year after detection of metastases. Bottom right: a plot indicating the cumulative score for Pin1, FOXM1, CENPF and Cyclin B1 (number of individual markers above population mean for each mRNA) vs disease outcome. Note that not all markers may be individually elevated in the group dead <1 year vs alive >2 year, but their cumulative score appears to have prognostic value. (**d** and **e**) Immunocytochemical staining for Pin1 and CENPF on sequential sections from a mouse skin of BRAF^V600E^-inducible mice that spontaneously develop melanomas in time.^23^ In the same animal, Pin1 and CENPF are barely expressed in the benign nevus (**d**), but are strongly elevated in a similar, sequential region the adjacent melanoma (**e**). (**f**) Immunocytochemical staining for Pin1 and CENPF in normal skin and a stage III human melanoma. Note that a different Pin1 antibody (mouse-derived) is used than in (**e** and **f**). Right panel; cyclin B1 staining in skin vs melanoma samples. **P<0.01.

Pin1 is a phospho-specific peptidyl-prolyl isomerase that induces a *cis*-to-*trans* conversion of peptide backbones,^18^ thereby exposing covered residues and allowing additional regulation of substrate activity.^19^ Pin1 facilitates substrate isomerization through interaction with phosphorylated Ser/Thr-Pro motifs. During cell cycle progression, FOXM1 is progressively phosphorylated on such motifs.^20^ We therefore expanded our database search by analyzing whether, in addition to FOXM1 gene expression, Pin1 correlates with FOXM1 activity in melanoma. CENPF and Cyclin B1 are two important FOXM1 targets that mediate the mitosis-regulatory effects of FOXM1.^20, 21^ Furthermore, FOXM1 and CENPF were recently shown to have a synergistic interaction that drives cancer malignancy.^22^ Therefore, we used CENPF and Cyclin B1 as markers for FOXM1 activity. Individual melanomas from independent data sets showed that FOXM1, CENPF and Cyclin B1, but not actin (control), significantly correlated with Pin1 expression (Figures 2a and b). Patients bearing tumors expressing high levels of Pin1, FOXM1, CENPF and Cyclin B1 showed a markedly higher mortality rate (Figure 2c), suggesting these markers may be applicable as a prognostic tool for disease outcome.

To determine whether the increased mRNA levels result in increased protein expression, we stained samples from a BRAF^V600E^-inducible mouse model that spontaneously develops melanomas,^23^ for Pin1 and FOXM1. Whereas the BRAF^V600E^-induced nevus showed only background staining for Pin1 and FoxM1, both were strongly elevated in a BRAF^V600E^-derived melanoma (Supplementary Figure A). We obtained the best staining on mouse tissues using two rabbit-derived antibodies against Pin1 and CENPF. Instead of performing co-staining, we therefore stained sequential sections of a nevus and a derived tumor. Although the nevus again only revealed background levels of Pin1 expression, Pin1 was elevated in the adjacent melanoma. Importantly, CENPF expression was elevated in the same area, indicating that in the Pin1-positive melanoma FOXM1 signaling is active (Figures 2d and e). Subsequently, we determined to what extent these results hold true in humans, by using a patient-derived skin and melanoma sample in which we were able to perform direct co-staining using a mouse-Pin1 antibody. Although the normal skin control failed to show significant staining of Pin1 and CENPF, these were both elevated the melanoma, as seen in the mouse situation and they strongly co-expressed within the same cells (Figure 2f and Supplementary Figure B). A separate staining for Cyclin B1 also revealed this FOXM1 target to be elevated (Figure 2f). Thus, Pin1 correlates both with FOXM1 expression itself and FOXM1 activity in melanoma and high Pin1-FOXM1 levels suggest a poor prognosis.

### Pin1 is essential for FOXM1 activity

**Figure 3:**
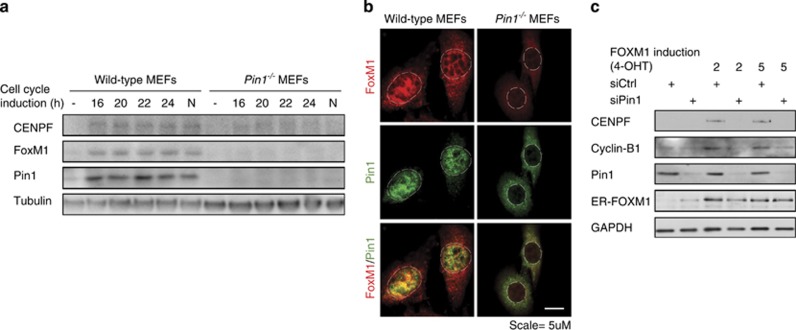
Pin1 is required for FOXM1 activity. (**a**) Expression of FOXM1 and CENPF is Pin1-dependent. Immunoblot analysis of the indicated proteins from lysates of wild type and *Pin1^−^*^*/−*^ MEFs synchronized in G0 by serum deprivation or induced to enter cell cycle progression by 10% FCS for the indicated intervals. *N*=Nocodazole-induced G2/M arrest. (**b**) *Pin1*^*−/−*^ MEFs show reduced FOXM1 expression under basal conditions. Immunocytochemical detection of Pin1 and FOXM1 in proliferating wild type and *Pin1*^*−/−*^ MEFs. The dashed lines indicate the contours of the nuclei. A merged image is shown to compare co-localization. (**c**) The induction of the FOXM1 target genes CENPF and Cylin-B1 by 4-OHT activatable FOXM1-ER is Pin1-dependent. U2OS cells stably expressing FOXM1-ER were transfected with control siRNA (siCtrl) or siRNA against Pin1 (siPin1). After 24 h they were exposed to 2 or 5μM 4-OHT and 24 h later processed for immunoblot analysis of the indicated proteins. Note that the ER-FOXM1 band is not significantly reduced by Pin1 knockdown, indicating stabilization due to the ER fragment.

Our correlation data raised the possibility that FOXM1 signaling is under direct control of Pin1. To address this, we determined whether Pin1 regulates FOXM1 activity. As both proteins are active during normal cell cycle progression, we first addressed whether Pin1 regulates FOXM1 under those conditions. FOXM1 expression is minimal in non-proliferating cells.^20^ On cell cycle initiation, FOXM1 is gradually stabilized and its transcriptional activity increases. This results in a peak in FOXM1 expression and activation during the G2/M phase of the cell cycle, followed by rapid degradation on mitotic exit. To determine whether Pin1 regulates FOXM1 activity during cell cycle progression, we induced cell cycle arrest by growth factor withdrawal in wild-type or *Pin1*^*−/−*^ mouse embryonic fibroblasts (MEFs) and subsequently followed how growth factor-induced cell cycle initiation affected the expression of FoxM1 and CENPF. FoxM1 and CENPF expression increased over the ensuing 24 h in wild-type MEFs as expected, but only marginally increased in *Pin1*^*−/−*^ MEFs (Figure 3a). Single-cell analysis confirmed that under proliferative conditions, FoxM1 is present in wild-type, but only marginally in *Pin1*^*−/−*^ MEFs (Figure 3b).

To further address the importance of Pin1 for FOXM1 activity, we next used an established cell line that stably expresses a chimeric FOXM1 protein fused to the ligand-binding domain of the estrogen receptor (ER).^20^ The resulting FOXM1-ER is inactive until stabilized by 4-hydroxytamoxifen, allowing for target gene expression. Indeed, 4-hydroxytamoxifen significantly elevated the expression of FOXM1-ER and its target genes Cyclin B1 and CENPF (Figure 3c). Strikingly, Pin1 depletion by RNA interference abrogated 4-hydroxytamoxifen-induced expression of CENPF and Cyclin B1. Consistent with the results obtained using *Pin1*^*−/−*^ MEFs, this indicates that Pin1 is essential for FOXM1 activity and supports a causal link between elevated Pin1 and FOXM1 activity in the melanoma samples of Figure 2.

### Pin1 regulates FOXM1 through MEK-dependent physical interaction

During cell cycle progression, FOXM1 is gradually phosphorylated on multiple residues, including the Ser-Pro motifs S331 and S704.^11^ Phosphorylation of these residues was shown to be MEK-dependent, but it remains unaddressed whether this is mediated by MEK itself or its target ERK1/2. We observed recombinant ERK1 to be able to phosphorylate FOXM1 *in vitro*, suggesting S331 and S704 are ERK target sites, phosphorylated in a MEK-dependent manner (Supplementary Figure C).

**Figure 4:**
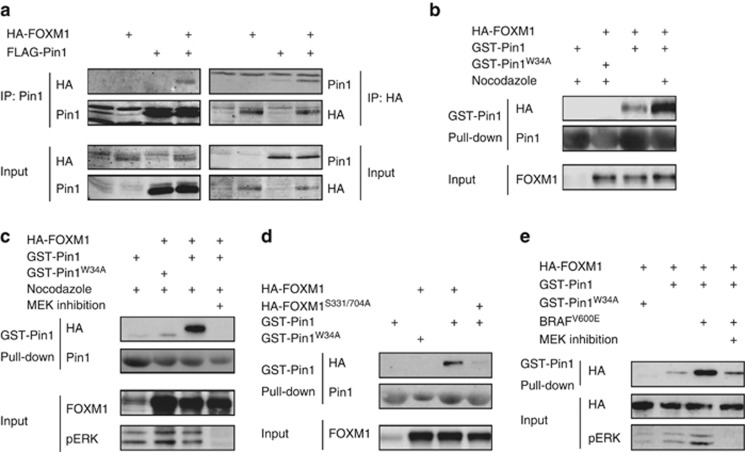
Pin1 physically regulates FOXM1 in a MEK-dependent manner. (**a**) Pin1 and FOXM1 physically interact. FLAG-Pin1 and HA-FOXM1 were transiently expressed in U2OS cells (input) and subjected to immunoprecipitation (IP) with anti-HA or anti-FLAG antibodies. Immunoblot analysis was performed with antibodies against Pin1 or HA to detect FOXM1. (**b**) The Pin1-FOXM1 interaction increases at the G2/M boundary of the cell cycle. U2OS cells transiently expressing HA-FOXM1 were treated for 24h with 250ng/ml Nocodazole to synchronize them at G2/M and lysates were subjected to a pull-down using recombinant GST-Pin1 or the substrate binding-deficient GST-Pin1^W34A^. Immunoblot analysis was performed as in **a**. (**c**) The Pin1-FOXM1 interaction is MEK-dependent. U2OS cells were treated as in **e**, but in the presence or absence of a 24 h pretreatment with a MEK inhibitor (10 μM U0126). (**d**) The MEK-target sites, S331 and S704, are essential for the Pin1-FOXM1 interaction. U2OS cells expressing wild-type HA-FOXM1 or a mutant in which Ser331 and Ser704 are mutated to Ala (HA-FOXM1^S331/704A^) were processed as described in (**b**). (**e**) BRAF^V600E^ stimulates the Pin1-FOXM1 interaction through MEK-dependent modification of FOXM1. U2OS cells expressing HA-FOXM1 and BRAF^V600E^ were left untreated or treated with for 24 h with a MEK inhibitor (20 μM PD184352) and processed as in (**b**).

Interference with MEK activity or mutation of the MEK/ERK target sites impairs the ability of FOXM1 to transactivate target genes and entry into mitosis.^11^ As Ser/Thr-Pro sites are potential Pin1-docking sites, we asked whether Pin1 can physically interact with FOXM1. Before addressing this for endogenous proteins, we first performed interaction assays using ectopically expressed epitope-tagged proteins. Under basal conditions, FOXM1 and Pin1 could be reciprocally co-immunoprecipitated (Figure 4a). Ectopically expressed FOXM1 could furthermore be pulled down using bacterially produced, recombinant Glutathion-S-Transferase (GST)-tagged Pin1, suggesting a direct interaction (Supplementary Figure D).

Pin1 interacts with substrates through recognition of phosphorylated Ser/Thr-Pro motifs by its WW-domain.^18^ To better understand the nature of the Pin1-FOXM1 interaction, we asked whether FOXM1 phosphorylation is required. Mutation of the critical Pin1 phospho-binding residue W34 abrogated the interaction (Supplementary Figure D). Moreover, post-lysis dephosphorylation of FOXM1 by λ-phosphatase attenuated its interaction with recombinant GST-Pin1 (Supplementary Figure E). These data identify FOXM1 as a phospho-specific substrate of Pin1.

Next, we addressed under which physiological conditions Pin1 interacts with FOXM1. FOXM1 is phosphorylated by MEK/ERK at the G2/M transition of the cell cycle to facilitate mitotic entry and execution.^11^ To determine whether MEK/ERK-dependent phosphorylation of FOXM1 at this cell cycle stage is required for its regulation by Pin1, we exposed the cells to Nocodazole, which arrests cells in G2/M by impairing the microtubule polymerization required for mitotic entry.^20^ FOXM1 extracted from G2/M-arrested cells was a significantly stronger interaction partner for GST-Pin1 than FOXM1 extracted from asynchronous cells (Figure 4b). Importantly, this effect was lost upon chemical inhibition of MEK (Figure 4c), indicating that MEK-mediated phosphorylation at the G2/M boundary primes FOXM1 for Pin1 binding. In addition, we generated a FOXM1 mutant in which both MEK-target sites Ser331 and Ser704 are mutated to Alanine, FOXM1^S331/704A^. In contrast to wild-type FOXM1, recombinant GST-Pin1 failed to precipitate this mutant (Figure 4d). Thus, Pin1 binds FOXM1 on its MEK/ERK target sites at the G2/M boundary of the cell cycle.

As MEK is frequently hyperactivated in melanomas due to oncogenic BRAF signaling, we also determined the effect of oncogenic BRAF on Pin1-FOXM1 binding. Interestingly, BRAF^V600E^ significantly increased the interaction in a fashion that was abrogated on chemical MEK inhibition (Figure 4e). Altogether, these experiments indicate that (I) Pin1 correlates with FOXM1 expression and activity in malignant melanoma, (II) Pin is causally linked to FOXM1 activity through physical binding on MEK/ERK target sites and (III) oncogenic BRAF stimulates the Pin1-FOXM1 interaction through MEK. This led us to address whether Pin1-FOXM1 signaling can be exploited for therapeutic intervention of BRAF^V600E^-driven melanomas.

### Pin1 inhibition represses FOXM1 activity and growth of metastatic BRAF^V600E^-driven melanoma cells

**Figure 5:**
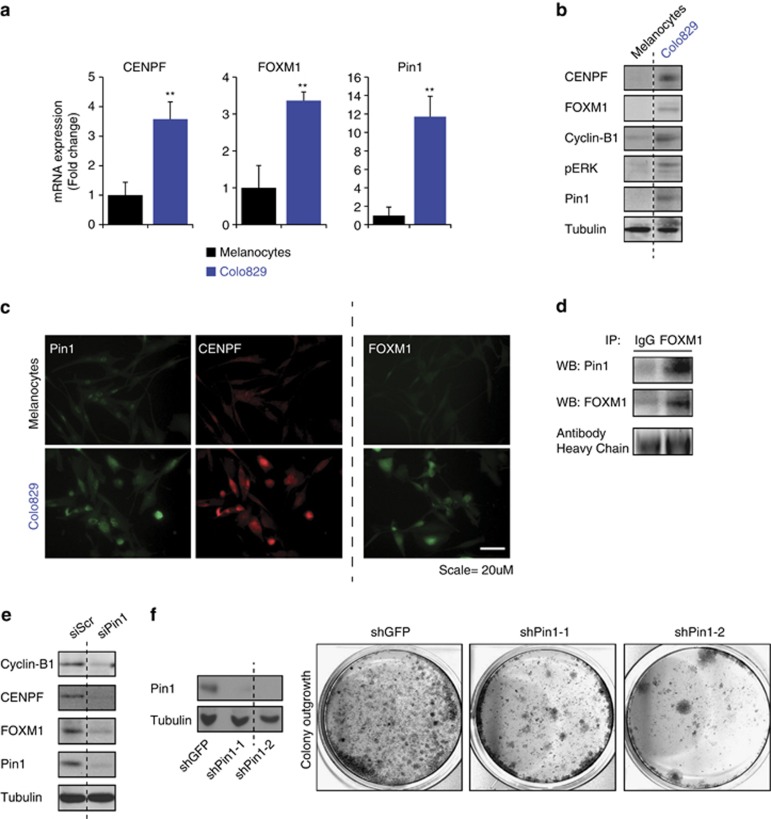
Pin1 is required for FOXM1 activity and melanoma proliferation in an endogenous BRAF^V600E^ background. (**a**–**c**) Colo829 melanoma cells express elevated levels of Pin1, FOXM1, CENPF and Cyclin B1. (**a**) QPCR-based detection of the indicated mRNAs in primary neonatal Human Epidermal Melanocytes (HEMn) and Colo829 melanoma cells. (**b**) Immunoblot detection of the indicated proteins corresponding to the mRNAs interrogated in (**a**). (**c**) Detection of the indicated proteins interrogated in **b** by immunofluorescence. (**d**) Endogenous Pin1 and FOXM1 interact in Colo829 cells. Co-immunoprecipitation (IP) of Colo829 lysates (Input) using an antibody against FOXM1 or control IgG. The antibody heavy chains show equal amounts of FOXM1 and control IgG antibodies were used. (**e**) Pin1 is essential for FOXM1 expression and activity. Colo829 cells were transfected with a control siRNA (siCtrl) or siRNA against Pin1 (siPin1) and processed for immunoblot analysis of the indicated proteins. (**f**) Prolonged Pin1 depletion represses Colo829 outgrowth. Colo829 were stably transduced with two independent lentivirally delivered shRNAs targeting Pin1 (shPin1) or a control shRNA (shGFP) and colony formation was addressed 7 days later. **P<0.01.

To investigate the importance of the elevated Pin1-FOXM1 signaling in melanoma, we searched for a representative cell line that recapitulates these elevations. Compared with neonatal human epidermal melanocytes, BRAF^V600E^-mutated Colo829 human melanoma cells showed enhanced expression of Pin1, FOXM1, CENPF and Cyclin B1 both at the mRNA and protein levels (Figures 5a and b). Similar to the human and mouse melanoma samples (Figures 2d and f) and the MEF samples (Figure 3b), Pin1 and CENPF co-expressed in individual Colo829 cells (Figure 5c). Also, endogenous Pin1 and FOXM1 co-immunoprecipitated in lysates from these cells (Figure 5d), indicating an endogenous Pin1-FOXM1 interaction.

To determine the role of endogenous Pin1-FOXM1 signaling in melanoma cells, we first depleted FOXM1. This resulted in a marked decrease in colony-forming potential (Supplementary Figure F), indicating that FOXM1 is important for the proliferative potential of Colo829. We also transiently repressed Pin1 expression in these cells (Figure 5e). Consistent with our results in the *Pin1*^*−/−*^ MEFs and ER-FOXM1-overexpressing cells (Figures 3a and c), Pin1 inhibition reduced the expression of endogenous FOXM1, CENPF and Cyclin B1 in Colo829 (Figure 5e). As Pin1 depletion might arguably suppress cell cycle progression, which regulates FOXM1 activity, we determined the effect of Pin1 depletion on FOXM1 activity in Colo829 cells arrested in G1/S by Thymidine or G2/M by Nocodazole.^20^ CENPF expression was detectable in G2/M arrested, but not G1/S arrested, cells (Supplementary Figure G), showing that, although being highly expressed under basal conditions, CENPF expression in Colo829 is still cell cycle-dependent. Notably, although transient knockdown of Pin1 did not affect the amount of G2/M-arrested Colo829 cells by Nocodazole, it strongly reduced CENPF expression. The effects of Pin1 inhibition on reduced FOXM1 activity are therefore independent of potential changes in cell cycle progression caused by Pin1 depletion. Encouraged by these results, we wondered what would be the effect of Pin1 inhibition on the malignant proliferation of these cells. As small interfering RNA is only effective for short periods of time, we applied stable knockdown of Pin1 using lentiviral small hairpin RNAs. Strikingly, prolonged repression of Pin1 markedly decreased the colony-forming potential of Colo829 (Figure 5f). Together these findings suggest that interference with the Pin1-FOXM1 interaction can be effective against melanoma progression.

### Cell-permeable Pin1-FOXM1-blocking peptides repress FOXM1 activity and melanoma cell viability

**Figure 6:**
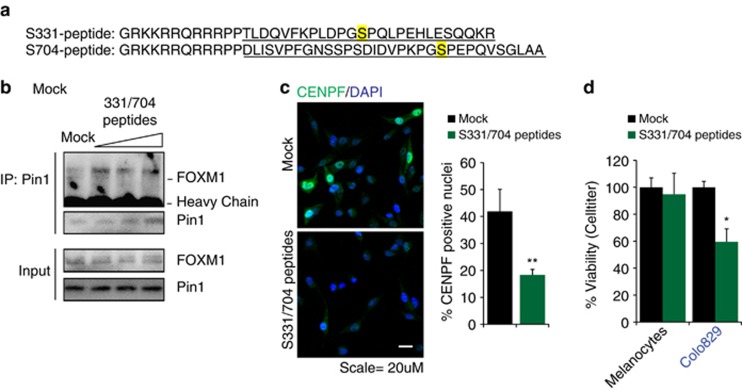
Pin1-FOXM1-blocking peptides repress FOXM1 activity and BRAF^V600E^-driven melanoma cell survival and colony formation. (**a**) Sequence of the S331 and S704 Pin1-FOXM1-blocking peptides. The MEK/ERK-phosphorylatable Pin1-binding sites S331 and S704 are highlighted in yellow. The FOXM1-mimicking sequences in the peptides are underlined. The amino acids GRKKRRQRRRPP comprise the TAT sequence of HIV, used to ensure cell permeability.^24^ Two prolines separate the two peptide sequences. (**b**) A mixture of the S331 and S704 peptides represses the interaction between endogenous Pin1 and FOXM1. Colo829 cells were treated for 24 h with 5, 10 and 25 μM of each peptide in a 1:1 ratio and lysates (Input) were subjected to immunoprecipitation (IP) using a Pin1 antibody and immunoblot analysis using antibodies against Pin1 or FOXM1. (**c**) The mix of S331/704 peptides reduces CENPF expression. Colo829 cells were treated with 25 μM of each of the 331 or 704 peptides in a 1:1 ratio and CENPF immunostaining was performed. The percentage of cells with nuclear CENPF signal was quantified using Cellprofiler software. **P<0.01. (**d**) The mix of S331/704 peptides reduces viability of Malignant Colo829 melanoma cells, but not of neonatal Human Epidermal Melanocytes as determined via the AqueousOne Solution Cell Proliferation Assay. *P<0.05.

To investigate whether the Pin1-FOXM1 interaction could be targeted for therapeutic use, we developed a set of peptides designed to interfere with the Pin1-FOXM1 interaction. These peptides mimic the sequences in FOXM1 neighboring the Pin1-binding sites S331 and S704 defined in Figure 4d. We rendered the peptides cell-permeable by fusion to the HIV TAT sequence^24^ (Figure 6a). Treatment of Colo829 with the S331/704 peptides resulted in decrease in the endogenous Pin1-FOXM1 interaction (Figure 6b), indicating that targeting the Pin1-FOXM1 interaction in melanoma cells is feasible.

Next, we investigated the effect of these peptides on FOXM1 activity and the concomitant malignant proliferation. Incubation of Colo829 with the Pin1-FOXM1-blocking peptides decreased CENPF expression (Figure 6c), indicating decreased FOXM1 activity. In turn, the Pin1-FOXM1-blocking peptides reduced the percentage of viable Colo829 melanoma cells but not normal melanocytes (Figure 6d). These results suggest that inhibition of the Pin1-FOXM1 interaction may be applicable against human melanoma and provided a stepping stone for testing the efficacy of these peptides in a more physiologically relevant setting using fresh patient-derived material.

### Direct Pin1-FOXM1 inhibition represses metastatic tumor progression in patient-derived melanoma slices *ex vivo* and melanoma cell viability in 3D-cultured melanoids

As cell lines have typically been in culture for prolonged periods of time, they have become adapted to tissue culture conditions and differ from the original situation in human patients. We therefore sought alternative models that better recapitulate this setting to test the efficacy of our Pin1-FOXM1-blocking peptides. Unfortunately, sequence alignment of human and mouse FOXM1 around the sequence of our peptides showed the S704 to poorly align with mouse FOXM1 (Supplementary Figure H), arguing against their use in experiments in spontaneous BRAF^V600E^-induced melanoma in mice such as the model described in Figures 2d and e. To overcome these limitations, we therefore used two independent techniques using fresh patient material, the first based on *ex vivo* culture of melanoma metastases and the second on three-dimensional (3D)-cultured melanoma organoids, here dubbed melanoids.

**Figure 7:**
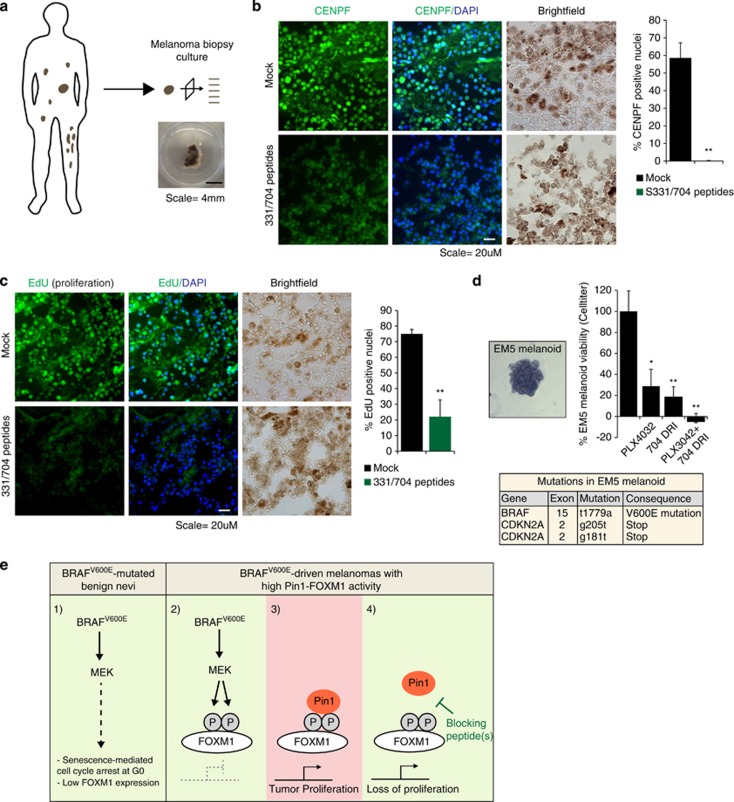
Pin1-FOXM1-blocking peptides repress FOXM1 activity and malignant proliferation in fresh human melanoma biopsy material. (**a**–**c**) The S331/704 peptides repress CENPF expression and malignant proliferation of a human metastatic melanoma biopsy. A melanoma was isolated, divided in 300-μM sections and either directly treated with the S331/704 peptides as in Figure 6c or left untreated. After 6 days, one set was processed for CENPF (**b**) immunostaining and quantified as in Figure 6c. Another set was incubated with EdU on day 5, fixed 24 h later and processed for EdU incorporation (**c**), which was quantified similarly. **P<0.01. (**d**) The FOXM1-S704 D-Retro-Inverso (DRI) peptide reduces viability of a patient-derived 3D-cultured melanoid, EM5. A node-resected Stage III metastatic melanoma was sectioned and cultured in Matrigel as in **a**. Subsequent passaging resulted in monocultures (left panel) with >96% BRAF^V600E^ and CDKN2A mutations as determined by Next Generation sequencing (bottom table and Supplementary Figure k). The melanoid cultures, dubbed ErasmusMC melanoma #5, EM5, were split into in 96-well plates and exposed to 0.25 μM PLX4032, 100 μM of the S704 DRI peptide or their combination. Cell viability determined 4 days later by AqueousOne Celltiter Assay (top right panel). (**e**) Model of the regulation of FOXM1 by Pin1 in BRAF^V600E^-driven melanoma. (1) Active BRAF^V600E^ induces a senescence-associated G0-based growth arrest resulting in low FOXM1 expression. (2) In melanomas where this arrest is escaped, FOXM1 becomes elevated and MEK-dependent FOXM1 phosphorylation occurs in the G2/M phase of the cell cycle. (3) MEK-mediated phosphorylation allows Pin1 binding, ensuring full FOXM1 activity and proliferation. (4) Interference with Pin1 or the Pin1-FOXM1 interaction by (**a**) specific blocking peptide(s) represses FOXM1 activity and proliferation, providing a potential point of intervention for melanomas expressing high levels of Pin1-FOXM1 signaling. *P<0.05; **P<0.01.

For *ex vivo* melanoma metastasis culture, a stage IV metastatic melanoma was sectioned in 300 μM thin slices, which were individually placed in culture media (Figure 7a), similar to what we previously showed for mammary carcinoma.^25^ The slices were subdivided for either vehicle treatment or incubation with the Pin1-FOXM1-blocking peptides. After 6 days the proliferative potential of the cells inside the tumor material was determined by measuring nuclear CENPF positivity and 24 h EdU incorporation. Excitingly, both CENPF staining and EdU incorporation were markedly reduced in the melanoma sections incubated with the Pin1-FOXM1-blocking peptides (Figures 7b and c). In line with the results showing that Pin1 regulates FOXM1 levels in culture (Figures 3a and 5e) also FOXM1 expression was repressed in these sections (Supplementary Figure I). Encouraged by these results, we sought to further extrapolate these data. A major benefit of melanoma tumor slice culture is these largely preserve the complexity and tumor-stromal interactions of the original tumor. A downside, however, is that they only allow for few conditions to be examined before the material is exhausted. To overcome this limitation, we therefore used part of the material for 3D organoid cultures, allowing for additional experiments to be performed on near-primary material of the same patients. To this extent we placed separate metastatic tumor sections in matrigel and allowed tumor outgrowth to occur. After removal of the primary tumor slice, clean melanoma cultures were obtained through multiple rounds of passaging to remove contamination of fibroblasts and other cell types (Figure 7d). Thus, we were able to use these cultures to test the effects of Pin1-FOXM1 inhibition on tumor growth.

So far, we used a combination of two independent peptides. Clinical translation of such a combination of two drugs is challenging as each will have to be separately tested for off-target effects. In our earlier experiments, we observed the S704 peptide, but not the S331 peptide, to show partial anti-melanoma activity on its own (not shown). To overcome this limitation of having to use a combination of peptides, we therefore sought to optimize the S704 peptide for use as single agent. To this extent, we designed a D-Retro-Inverso (DRI) version of the S704 peptide, which is potentially more effective, as was observed for other cell-permeable peptides.^26^ First, we tested the FOXM1-S704 DRI peptide on 3D melanoid cultures using two independent BRAF^V600E^-mutated cell lines, Colo829 and Malme-3M, both of which are sensitive to the BRAF^V600E^-inibitor PLX4032 (Vemurafenib; Supplementary Figure J). A single dose of this peptide reduced Colo829 and Malme-3M melanoid viability (Supplementary Figure J), prompting us to determine the effects of the peptide on melanoids grown straight from patient-derived material. To this extent, we generated a melanoid culture to faithfully mimic the majority of metastatic melanomas as described in Figures 1 and 2, ErasmusMC Melanoma #5 (EM5). EM5 was isolated from a stage III lymph node-resected metastatic melanoma that was TP53 wild type, BRAF^V600E^-mutated and CDKN2 A-deficient (Figure 7d and Supplementary Figure K), thereby passing these criteria. More potently than in the established cell lines, FOXM1-S704 DRI reduced the number of viable cells of this 3D melanoid culture (Figure 7d). As BRAF inhibition is potent against metastatic melanoma, but allows for recurrence in time, we ultimately set out to determine to what extent this peptide could be complementary to the clinically frequently prescribed BRAF^V600E^-inhibitor PLX4032, Vemurafenib. Excitingly, whereas both PLX4032 and the FOXM1-S704 DRI peptide individually reduced EM5 melanoid viability, their combination decreased viability to levels below background, indicating that FOXM1-S704 can be additive to BRAF inhibition against human melanoma (Figure 7e).

Together, these independent *ex vivo* assays, using fresh patient-derived metastatic melanoma slices or 3D melanoids, show that direct inhibition of the Pin1-FOXM1 interaction can be used to repress FOXM1 activity and malignant melanoma proliferation, the latter of which is applicable to complement BRAF inhibition. These results provide a starting point for further optimization and may open opportunities for therapeutic intervention, in particular, of high FOXM1-expressing metastatic melanomas.

## Discussion

To target metastatic melanoma, inhibitors of the RAF/MEK pathway have shown partial clinical success,^7, 8^ but their effectiveness is hampered due to development of resistance.^27^ To improve the prognosis for melanoma patients, efforts now focus on either intermittent treatment schedules for existing drugs,^28^ development of new RAF/MEK inhibitors,^29, 30^ sequential treatment of resistant tumors^31^ or combination therapies.^27, 32^ Much less attention has been devoted to targeting proteins that function downstream of MEK and promote cell survival or cell cycle progression. These potentially provide an uncharted field of targetable opportunities.

Here, we showed that many human melanomas express elevated levels of the pro-proliferative and pro-survival MEK-target FOXM1. Inhibition of its enzymatic regulator Pin1 or the Pin1-FOXM1 interaction successfully repressed FOXM1 activity and tumor proliferation *ex vivo* of in a freshly cultured human melanoma tumor and in 3D-cultured patient-derived melanoids (Figure 7). It should be noted that the effects in 3D were more robust than in 2D and more efficient in fresh patient-derived samples than in established cell lines. Potentially cell–cell interactions and cell–matrix interactions are therefore important to take into account. FOXM1 has been associated with epithelial-to-mesenchymal transition, E-Cadherin expression, migration and secretion of inflammatory proteins (for example, reviewed in^33^ all of which are more important in 3D, hence patients, than in 2D. We also addressed apoptosis in 2D, but this did not appear to be strongly affected by the FOXM1 peptides. Regardless of the cause, these results provide a proof-of-concept that Pin1-FOXM1-blocking peptides are functional against melanoma progression in a physiological setting *ex vivo* and are a therefore potentially applicable therapeutic tool to complement BRAF/MEK inhibitors to clinically target metastatic melanoma (Figure 7e). It will be interesting to see if further optimization of the peptides is possible by using additional modes of delivery, altered sequences or longer stretches of the peptide and whether these are applicable against BRAF/inhibitor-resistant tumor from patients.

In this study, we specifically focused on the role of Pin1-FOXM1 signaling in melanoma. It is tempting to speculate that FOXM1 inhibition might be beneficial to cancers other than melanoma. FOXM1 is not only elevated in melanoma, but also in other types of cancer (Supplementary Figure M). However, Pin1 expression does not appear to significantly correlate with FOXM1, CENPF and Cyclin B1 expression in at least mammary, ovarian, renal and endometrial carcinomas and glioblastoma (Supplementary Figure N), suggesting the regulation of FOXM1 activity by Pin1 might be restricted to subsets of cancers.

BRAF^V600E^ is not only active in melanoma, but also in benign nevi. In nevi, however, the CDK4/6 inhibitor p16^ink4a^ induces senescence and a strong G1/S arrest, which would restrain FOXM1 expression and its phosphorylation by MEK. Indeed, we observed low FOXM1 expression in nevi (Figure 2a, Supplementary Figure A). p16^ink4a^ is frequently lost in melanoma due to genetic ablation^15^ (Figure 1c), which would allow FOXM1 expression. FOXM1 recently gained interest as a target of the CDK4/6 complex that is inhibited by p16^ink4a^.^34^ Thus, it is possible that, due to the defective repression of CDK4/6 in p16^ink4a^-mutated cells, FOXM1 is initially stabilized by this complex, allowing the subsequent MEK-dependent phosphorylation and the recognition by Pin1 we observed here. Interestingly, FOXM1 has been identified in hepatocellular carcinoma as a target of Alternate Reading Frame of the INK4a/ARF locus (CDKN2A), which also encodes p16^ink4a^.^35^ Arg-loaded peptides designed to interfere with ARF activity showed to reduce FOXM1 expression. In melanomas, including the cell line used here, however, expression of the CDKN2A locus is frequently lost (see Figure 1c).^15^ This still results in excessive expression of FOXM1 in melanomas (Figures 2a and f) and the here used CDKN2A-deficient cells (Figures 5a and c), indicating that ARF1 expression is not critical to FOXM1 expression in CDKN2A-depleted melanomas and FOXM1 inhibition cannot be targeted this way. It may be interesting to determine whether the Pin1-FOXM1 interaction can be perturbed by CDK4/6 inhibitors such as PD0332991^34^ leading to FOXM1 destabilization. The Pin1-FOXM1 inhibitory peptides we designed in this study (Figure 6a and Figure 7d) do show inhibitory potential toward FOXM1 activity and strongly repress tumor growth in a human melanoma *ex vivo* and 3D-cultured melanoids, complimentary to PLX4032 and as single entities.

To study new anticancer therapies, cell lines are a frequent starting point because they allow many experimental conditions to be inexpensively and rapidly tested. It remains challenging, however, to recapitulate the complexity of tumors and interactions between malignant cells and stroma in culture. Mice can overcome this limitation, but present their own challenges. Faithfully mimicking natural development of melanoma in mice is still an ongoing task as there are notable differences in melanoma development in humans and mice and mice generally express less cutaneous melanocytes.^36, 37^ Recent advances have been made in generating inducible BRAF^V600E^ mice either in combination with PTEN loss.^38, 39^ Targeting proteins in mice, however, requires a high degree of sequence homology, which is sometimes absent. Also in case of our Pin1-FOXM1-targeting peptides the ability of targeting endogenous FOXM1 in mice is low due to imperfect sequence similarity (Supplementary Figure H). To overcome this limitation, we used two independent systems using fresh patient-derived material instead, melanoma slice culture and 3D melanoids. Melanoma slice culture allows multiple experimental conditions to be tested on material from a single patient, yet to a degree maintains the complexity and microenvironment of the original human tumor. Outcomes from experiments performed on such *ex vivo* cultured metastasis material may provide a direct functional answer on whether a treatment is likely to succeed in that patient without having to perform mouse xenografts studies or pharmacogenomics. As metastases generally share a degree of genetic similarity, results from experiments on *ex vivo* cultured material from a metastatic cancer patient could potentially also be translated to additional metastases in the same patient, thus providing a powerful tool for the development of patient-specific therapies. The 3D melanoids could also be used for prognostic use of drug sensitivity, but have an added benefit that they can be maintained for longer periods of time. Though typically ignored, results in 2D can differ from 3D. Though the FOXM1 peptide(s) did show certain effects in long cultured cell lines in 2D, the effects in primary tissue—either in slice culture or in melanoids, were more potent and more reproducible. Thus, initial experiments on simple individual melanoma slice cultures or 3D melanoids from a metastatic patient may predict whether that individual could benefit from Pin1-FOXM1 inhibitory therapy. At least a subset of melanoma patients display elevated Pin1-FOXM1 signaling (Figure 2a) in combination with a poor prognosis and for these patients Pin1-FOXM1-blocking peptides may be a new treatment option, either alone or in combination with RAF/MEK inhibitors (see Figure 7).

## Materials and methods

### Cell culture and transfection

All cells were maintained in Dulbecco's Modified Eagle's Medium, 10% fetal calf serum (FCS), penicillin/streptomycin and 0.05% glutamine. U2OS cells stably expressing 4-hydroxytamoxifen activatable FOXM1-ER were further cultured with 0.2 μg/ml puromycin. *Pin1^−/−^* MEFs were a kind gift of Dr P van der Sluijs (UMC Utrecht). HEK293T and U2OS cells were transfected using calcium-phosphate as described.^40^ Colo829 cells were transfected with effectene according to the manufacturer's protocol (Qiagen, Venlo, the Netherlands). Of note, when obtained fresh from the supplier (ATCC), the Colo829 cells contain many non-dividing senescent cells. We obtained the best results using Colo829 populations in the early log phase of proliferation. Similar effects hold true for Malme-3M (NCI, Rockville, MD, USA).

### Reagents

The following compounds were purchased: nocodazole (Sigma, St Louis, MO, USA), U0126 (Promega, Madison, WI, USA), PLX4032 (SelleckChem, Munich, Germany) and Matrigel (Corning, Corning, NY, USA). PD184352 was a kind gift of Dr Philip Cohen (King's College, London, UK).

### Antibodies

The HA antibody (12CA5) has been described.^40^ The following antibodies were purchased. Cell Signaling (Leiden, The Netherlands): phosphoThr202/Tyr204-ERK (9101), Santa Cruz Biotechnology (Heidelberg, Germany): BRAF (C19), Cyclin B1 for immunoblot (GNS1), FOXM1 for immunoprecipitation, immunoblot and immunofluorescence *in vitro* (MPP2; C-20 and H-92), control immunoglobulin G, R&D systems (Minneapolis, MN, USA): Pin1 (MAB2294), Sigma-Aldrich: Pin1 (WH0005300M1, St Louis, MO, USA), FLAG-M2 (F1804), Tubulin (T5168), Abcam: CENPF (Ab-5, Cambridge, UK) and FOXM1 for immunofluorescence in tissue (ab175798), Chemicon: GAPDH (AB2302, Merck Millipore, Billerica, MA, USA), Pierce: cyclin B1 for immunofluorescence (MA5-13128, Thermo Fisher Scientific, Waltham, MA, USA).

### Co-immunoprecipitation and GST-Pull down assays

Immunoprecipitations and GST-pull-down assays were performed in lysis buffer containing 20 mM Tris-HCl (pH 8.0), 1% TX-100, 0.5% NaDoC, 5 mM EDTA, 150 mM NaCl, protease and phosphatase inhibitors as described.^41^ Endogenous co-immunoprecipitations were performed using a lysis buffer containing 20 mM of Tris-HCl (pH 8.0), 1% NP40, 10% glycerol, 1 mM of MgCl_2_, 1 mm of EDTA, 150 mM of NaCl, protease and phosphatase inhibitors.

### Cell cycle distribution

U2OS cells were transfected with the appropriate plasmids in combination with 250 ng GFP-Spectrin. At 36 h post transfection cells were treated for an additional 24 h with nocodazole or thymidine and processed for fluorescence-activated cell sorting analysis as described^40^ on a FACScalibur (ABI), using WinMDI v2.9 to analyze the data.

### Quantitative real-time PCR

To detect mRNA expression, mRNA of three independent samples was extracted using the Cells-to-Ct kit (Ambion, Austin, TX, USA). Quantitative PCR was subsequently performed using the Universal Probe Library system (Roche, Branchburg, NJ, USA) with the following primer/probe combinations: CENPF-UPL74–forward: 5′-gagtcctccaaaccaacagc-3′, CENPF-UPL74–reverse: 5′-tccgctgagcaactttgac-3′, FOXM1-UPL11–forward: 5′-actttaagcacattgccaagc-3′, FOXM1-UPL11–reverse: 5′-cgtgcagggaaaggttgt-3′, Pin1-UPL1–forward: 5′-gaagatcacccggaccaag-3′, Pin1-UPL1–reverse: 5′-aagtcctcctctcccgactt-3′, and Tubulin-UPL58–forward: 5′-cttcgtctccgccatcag-3′ and Tubulin-UPL58–reverse: 5′-ttgccaatctggacacca-3′.

### Overexpression and RNA interference

The following constructs have been described: pcDNA3, pBabe-Puro, pEFm-BRAF^V600E^ and pSuperior-shScrambled,^40^ pcDNA3-FLAG-Pin1, pCDNA3-FLAG-Pin1^W34A^, pGEX-GST-Pin1 and pGEX-GST-Pin1^W34A^.^41^ pcDNA3-HA-FOXM1 was generated by ligation of an HA-FOXM1 fragment obtained by PCR using the oligonucleotides EcoRI-HA-FOXM1f: 5′ccgggatccatgtacccatacgatgttccagattacgctcttgccgaggcgcctcaggtgg3′ and EcoRI-FOXM1r: 5′ccggaattcctactgtagctcaggaataaactg3′ into the EcoRI site of pcDNA3 (Invitrogen, Waltham, MA, USA). Correct orientation was verified by sequencing. HA-FOXM1c^S331/S704A^ was created by two rounds of site-directed mutagenesis using the following oligonucleotides: S331A–forward: 5′-ccactggacccaggggctccacaattgcccg-3′ S331A–reverse: 5′-cgggcaattgtggagcccctgggtccagtgg-3′, S704A–forward: 5′-gtccccaagccaggcgccccggagccacagg-3′, S704A–reverse: 5′-cctgtggctccggggcgcctggcttggggac-3′).

siRNA against Pin1 or a scrambled sequence (Dharmacon, Lafayette, CO, USA) have been described^41^ and were transfected at a final concentration of 100 nM using Oligofectamine according to the manufacturer's protocol (Invitrogen).

Lentiviral particles containing small hairpin RNAs against Pin1 were generated using a standard third-generation packaging protocol with the pLK0.1-based plasmids TRCN0000001033 containing the mature coding sequence: 5′-CCACCGTCACACAGTATTTAT-3′ (shPin1-1) and TRCN0000049211 containing the mature coding sequence: 5′-CGGCTACATCCAGAAGATCAA-3′ (shPin1-2) from Open Biosystems. pLK0.1-shGFP containing the mature sequence 5′-GCAAGCTGACCCTGAAGTTCAT-3′ was used as a control.

### Colony formation assay, AqueousOne Solution Cell Proliferation Assay and TUNEL staining

Colo829 cells were stably transduced with the indicated lentiviral particles. The next day the media was refreshed and 2 days later again with media containing 2 μg/ml puromycin. Three days later, cells were refreshed once without puromycin. Twenty-four hours later equal numbers of cells were plated in triplicate in six-well plates for each condition. Seven days later, the cells were fixed in MeOH and visualized after staining with 1% crystal violet in 25% MeOH. In case of the S331/704 peptides, cells were similarly fixed and processed at 7 days after incubation with 50 μM of the mix. The CellTiter96 AqueousOne Solution Cell Proliferation Assay was performed according to the manufacturer's instructions (Promega) in a 96-well plate containing Colo829 cells treated on day 1 and 3 with 50 μM of the S331/704 peptide mix and measured at day 5. Terminal deoxynucleotidyl transferase dUTP nick end labeling staining was performed according to the manufacturer's instructions (Roche).

### Pin1-FOXM1-blocking peptides

Cell-permeable peptides designed to interfere with the Pin1-FOXM1 interaction were obtained from NeoBioSci and comprised the following sequences (see Figure 6a):

FOXM1-S331: GRKKRRQRRRPPTLDQVFKPLDPGSPQLPEHLESQQKR (S331 underscored) and FOXM1-S704: GRKKRRQRRRPPDLISVPFGNSSPSDIDVPKPGSPEPQVSGLAA (S704 underscored). The FOXM1-S704 DRI peptide has an identical amino-acid sequence as the regular S704 peptide, but in a DRI isoform and was ordered at >90% purity from PepScan (Lelystad, the Netherlands).

### Melanoma biopsy isolation and treatment

Informed consent was obtained for use of the tumor sample in this study. The human melanoma was excised and placed in a 50-ml conical tube containing Dulbecco's modified eagle medium 10% FCS. Subsequently, it was sliced in 300-μM sections using a Vibratome (Leica, Eindhoven, the Netherlands). The sections were maintained under gentle rocking (60 rpm) in a humidified incubator at 37 °C, 5% CO_2_ in Dulbecco's modified eagle medium 10% FCS. The biopsy sections were incubated with FOXM1-blocking peptides and 5 days later incubated with EdU. One day later, they were fixed and processed for immunofluorescence detection of CENPF or cytochemical detection of EdU according to the manufacturer's instructions (Life Technologies, Bleiswijk, the Netherlands).

### 3D-cultured melanoma cell line and patient-derived organoids (melanoids)

For 3D-cultured patient-derived melanoids, a previously untreated stage III human metastatic melanomas was resected from a lymph node and processed for biopsy culture as described above. Subsequently, sections were placed in Matrigel (Corning) in a 24-well plate and allowed to gellify for 5 min at 37 °C after which they were incubated with Dulbecco's modified eagle medium 10% FCS, Pen/Strep. Tumor cell outgrowth was allowed to occur and the primary tumor section was removed. After expansion, the melanoids were passaged through resuspension with a Pasteur pipet, followed by mild centrifugation in 15-ml tubes. Subsequently, the tubes were cooled and ice-thawed Matrigel (Corning) was added followed by plating in 24-well plates. This was continued for several rounds to eliminate non-melanoma cells. Next-generation sequencing as described,^42^ using a selected panel (Clo-TUM) of whole-gene mutation analysis, hotspot analysis and single-nucleotide polymorphisms analysis, showed BRAF^V600E^-mutation and CDKN2A mutation in >96% of the population, confirming the purity of the culture. For the experiments in Figures 7d and e, the resuspended melanoids were plated in 96-well plates, allowed to gellify and supplemented with growth media (Dulbecco's modified eagle medium, 10% FCS, Pen/Strep). The next day the melanoids were plated in quadruplo and incubated with the FOXM1 DRI and/or PLX4032 as indicated and cell viability was determined 36 days later using AqueousOne Celltiter assay (Promega).

For 3D-culture of established human melanoma cell lines, 1000 cells were placed in matrigel and allowed to form spheres in 24-well plates. When sufficiently grown, these were split in triplicate into 96-well plates in fresh Matrigel (2 μl/well). Following treatment with the FOXM1 DRI peptide or PLX4032 as indicated, cell viability was determined as above.

### Sequence alignments

To compare human and mouse FOXM1, the following sequences from National Center for Biotechnology Information were used: Human FOXM1: NP_973731, Mouse FOXM1: NP_032047.4.

### Statistical analysis of database studies

The data from study 1–6 were obtained from the Oncomine database: www.oncomine.org. For Figure 1a, the data from studies (43) and (44) were analyzed for differential expression of melanoma vs using Ingenuity Pathway Analysis; Version build 242990. Shown are those factors that have a predicted activation *Z*-score>2, are themselves upregulated more than twofold and are significantly different between skin and melanoma (*P-*value<0.05; Student's *t*-test). For Figures 1b, a differential expression analysis comparing melanomas isolated from primary sites to metastases was performed on the data from studies (44) and (45) analyzed using Student's *t*-test. For Figure 1c, the data from studies (46) and The Cancer Genome Atlas melanoma (unpublished) were analyzed by one sample *t*-test for foxm1 and cdkn2a gene copy numbers in melanoma and cutaneous melanoma, respectively. For Figure 1d data from studies (43) and (47) was processed for analysis of mRNAs that co-express with FOXM1. The resulting analysis was further processed in Ingenuity Pathway Analysis for 'upstream regulator' analysis. Shown are the top enzymes that co-express with FOXM1 and their correlation score (*r*^2^). For Figure 2a, data from (43, 44, 48) were analyzed for differential expression of Pin1, FOXM1, CENPF, Cyclin B1 (CCNB1) and actin (ACTA1), between the indicated groups. All values, except for actin, were significantly different between skin and melanoma (study 2+3) or nevi and melanoma (study 3 only). For Figure 2b, the *r*-values between the indicated mRNAs were determined to address their correlation. For Figures 2c, *z*-score corrected expression values of the indicated mRNAs were plotted and for each patient it was determined whether a score was higher or lower than the experimental mean. The number of markers higher than the experimental mean were plotter for each patient and plotted in survival <1year or >2year. Unpaired Student's *t*-test analysis was used to compare the two cohorts.

### Statistical analysis of experimental data

In all histograms, the s.d. is plotted, except for Celltiter AQueous One (Promega) assays, where s.e.m. is plotted. In all cases Student's *t*-tests were applied to compare significance between two groups. *P-value <0.05, **P-value <0.01.
