# Supplementary material for: Targeted inhibition of metastatic melanoma through interference with Pin1-FOXM1 signaling
Source: Oncogene. 2015 Aug 17;35(17):2166–77. doi: 10.1038/onc.2015.282 (PMC4757516; doi:10.1038/onc.2015.282)
Supplement: Supplementary Figures Legends [file onc2015282x1.docx]

Supplementary Figure legends

A) Pin1 and FoxM1 are elevated in a BRAF^V600E^-driven([1](#_ENREF_1)) mouse melanoma, but not a BRAF^V600E^-mutated nevus. Staining as in Fig. 2D+E, using Pin1 and FOXM1 antibodies.

B) Pin1 and FOXM1 are elevated in a Stage III human melanoma, but not normal skin. Shown are the individual Pin1 and FOXM1 stainings from Fig. 2F.

C) FOXM1 is a substrate for ERK1 *in vitro*. HA-FOXM1 (2μg) was transiently expressed in U2OS cells and immunoprecipitated using an anti-HA antibody. Subsequently, recombinant active ERK1 and 10μCi ^32^P-γ-ATP were added and phosphorylation detected by phosphoimaging. Total FOXM1 and recombinant ERK1 were additionally detected by western blot using the indicated antibodies.

D) The binding between Pin1 and FOXM1 is dependent on the WW-domain of Pin1. GST-pull down using bacterially produced recombinant GST-Pin1 or GST-Pin1^W34A^ and lysates from HA-FOXM1 transfected cells.

E) The interaction between FOXM1 and Pin1 is dependent on phosphorylation of FOXM1. Experiment as in D), but with the HA-FOXM1 containing lysate treated or not with λ-phosphatase (λ-ppase) to induce protein dephosphorylation.

F) FOXM1 knockdown represses Colo829 colony forming potential. Colo829 were transfected with either shScr control or shFOXM1 and processed for colony formation. In parallel immunoblot samples were generated to analyze knockdown efficiency.

G) Pin1 is required for FOXM1 expression and activity independent of cell cycle position. Colo829 were synchronized in G1/S by Thymidine or G2/M by Nocodazole and processed as in 5E). Cell cycle distribution profiles are shown to verify the effects of the Thymidine and Nocodazole blocks in siCtrl and siPin1 transfected Colo829 prior to and after removal.

H) The Pin1-FOXM1 targeting peptide S704 poorly aligns with mouse FOXM1. Alignment of the human FOXM1 sequence used for the S704 peptide (S704 indicated in yellow) with mouse FOXM1. This peptide would unlikely be able to target mouse FOXM1.

I) The S331 and S704 Pin1-FOXM1 blocking peptides reduce FOXM1 expression in an *ex vivo* cultured human metastatic melanoma. Shown are slices from the same experiment as in Fig. 7A-C, but stained for FOXM1.

J) The S704 DRI peptide reduces viability of two independent melanoids from BRAF^V600E^-driven, PLX4032-sensitive cell lines Colo829 and Malme-3M. Colo829 and Malme3M cells were allowed to form 3D melanoids and exposed to a single dose of 0.25uM PLX4032 or 100μM S704 DRI peptide. Cell viability was determined after 6 days using AqueousOne Celltiter assay.

K) Table showing non-mutated genes, cancer hotspots and SNPs in EM5 monocultures. Mutational analysis through Next Generation Sequencing as in Fig. 7D).

L) FOXM1 expression is strongly elevated in melanoma. Gene expression analysis on a dataset from the Oncomine database (Ramaswamy Multi-Cancer([2](#_ENREF_2))) comparing FOXM1 expression across different types of cancer.

M) The correlation in gene expression between Pin1 and FOXM1, CENPF and Cyclin B1 observed for melanoma (Fig. 2A+B) is not conserved in mammary carcinoma, glioblastoma, renal, ovarium and endometrial carcinoma. Database analysis on gene expression data from the Oncomine database using the indicated studies([3-6](#_ENREF_3)). Analysis as in Fig. 2B, green represents a correlation of R>0.45; p<0.01.

1. Dhomen N, Reis-Filho JS, da Rocha DS, Hayward R, Savage K, Delmas V, et al. Oncogenic Braf induces melanocyte senescence and melanoma in mice. Cancer Cell. 2009;15(4):294-303.

2. Ramaswamy S, Tamayo P, Rifkin R, Mukherjee S, Yeang CH, Angelo M, et al. Multiclass cancer diagnosis using tumor gene expression signatures. Proc Natl Acad Sci U S A. 2001 Dec 18;98(26):15149-54.

3. Curtis C, Shah SP, Chin SF, Turashvili G, Rueda OM, Dunning MJ, et al. The genomic and transcriptomic architecture of 2,000 breast tumours reveals novel subgroups. Nature. 2012 Jun 21;486(7403):346-52.

4. Gluck S, Ross JS, Royce M, McKenna EF, Jr., Perou CM, Avisar E, et al. TP53 genomics predict higher clinical and pathologic tumor response in operable early-stage breast cancer treated with docetaxel-capecitabine +/- trastuzumab. Breast Cancer Res Treat. 2012 Apr;132(3):781-91.

5. Sun L, Hui AM, Su Q, Vortmeyer A, Kotliarov Y, Pastorino S, et al. Neuronal and glioma-derived stem cell factor induces angiogenesis within the brain. Cancer Cell. 2006 Apr;9(4):287-300.

6. Murat A, Migliavacca E, Gorlia T, Lambiv WL, Shay T, Hamou MF, et al. Stem cell-related "self-renewal" signature and high epidermal growth factor receptor expression associated with resistance to concomitant chemoradiotherapy in glioblastoma. J Clin Oncol. 2008 Jun 20;26(18):3015-24.
