# Supplementary material for: Targeted inhibition of metastatic melanoma through interference with Pin1-FOXM1 signaling
Source: Oncogene. 2015 Aug 17;35(17):2166–77. doi: 10.1038/onc.2015.282 (PMC4757516; doi:10.1038/onc.2015.282)

Supplementary Figures

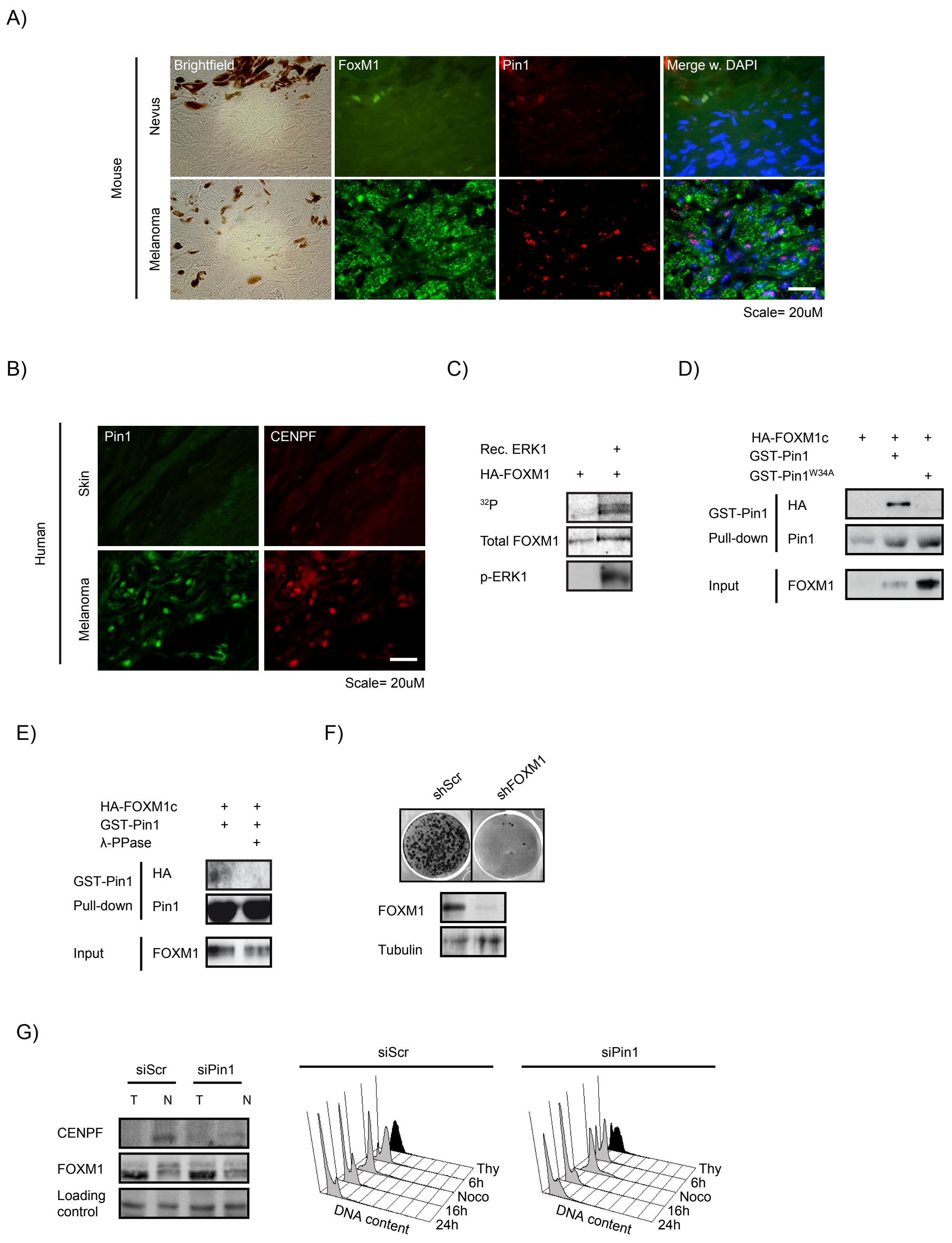

Supplementary Figures

H)

```
Human pldlisvpfgnsspsdidvpkpgspepqvsglaa
      |.||.|.|||:..|..::.|||||||.|:..|:|
Mouse pfdlasdpfgspppphvegpkpgspelqipslsa
```

I)

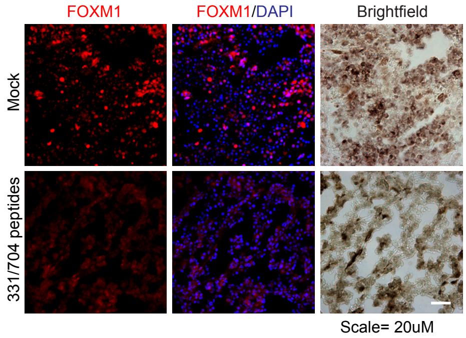

J)

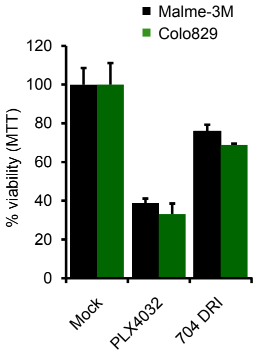

K)

Non-mutated sequences in EM5 melanoid
Whole genes:<br>PTEN, p53
Cancer hotspots:<br>AKT1, ALK, APC, CTNBB1, EGFR, ERBB2, EZH2, FBWX7, FOXL2, GNA11, GNAQ, GNAS, HRAS, IDH1, IDH2, KIT, KRAS, MET, NOTCH1, NRAS, PDGFRa, PIK3CA,RET, SMAD4, STK11
SNPs: 1p, 8p, 19q, APC, ARID1A, ATM, BRCA1, BRCA2, CDKN2A, FHIT, PTEN, RB1, SMAD4, STK11, TP53, VHL

L)

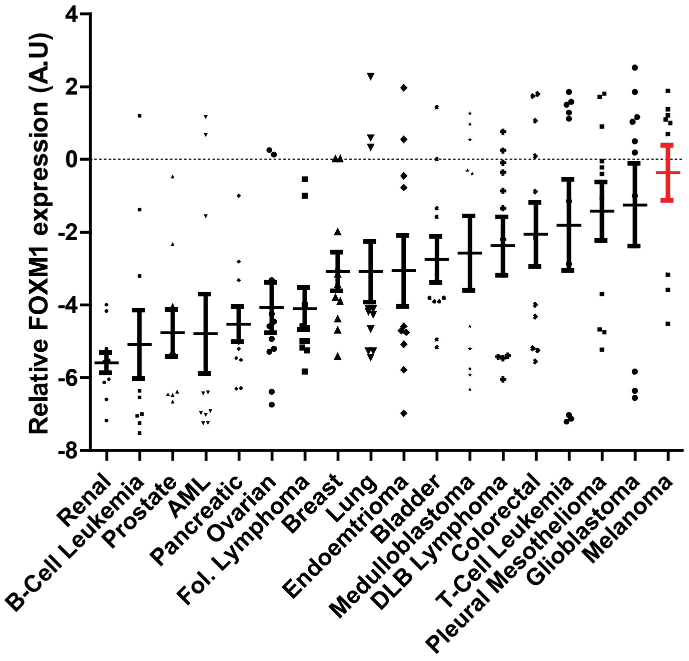

M)

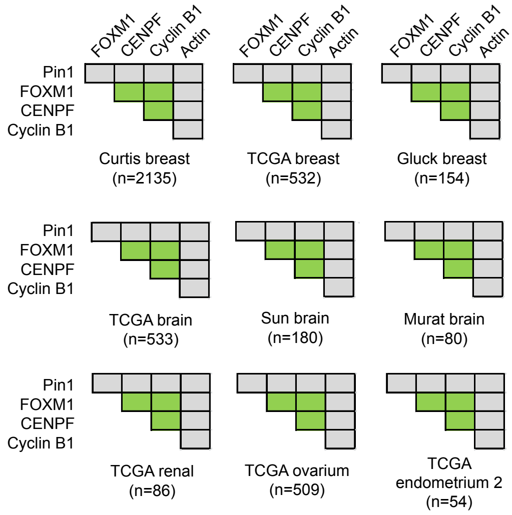

Supplement: Supplementary Figures [file onc2015282x2.pdf]
